# Functional Characterization of Cardiac Actin Mutants Causing Hypertrophic (p.A295S) and Dilated Cardiomyopathy (p.R312H and p.E361G)

**DOI:** 10.3390/ijms23084465

**Published:** 2022-04-18

**Authors:** Roua Hassoun, Constanze Erdmann, Sebastian Schmitt, Setsuko Fujita-Becker, Andreas Mügge, Rasmus R. Schröder, Matthias Geyer, Mina Borbor, Kornelia Jaquet, Nazha Hamdani, Hans Georg Mannherz

**Affiliations:** 1 Institut für Forschung und Lehre (IFL), Molecular and Experimental Cardiology, Medical Faculty, Ruhr University Bochum, D-44791 Bochum, Germany; roua.hassoun@rub.de (R.H.); andreas.muegge@ruhr-uni-bochum.de (A.M.); kornelia.jaquet@rub.de (K.J.); 2 Department of Cardiology, St. Josef-Hospital, Medical Faculty, Ruhr University Bochum, D-44791 Bochum, Germany; 3 Department of Anatomy and Molecular Embryology, Medical Faculty, Ruhr-University Bochum, D-44780 Bochum, Germany; constanze.erdmann@rub.de; 4 Institute of Structural Biology, University of Bonn, D-53127 Bonn, Germany; sebastian.schmitt@gmx.at (S.S.); matthias.geyer@uni-bonn.de (M.G.); 5 Cryoelectron Microscopy, BioQuant, Medical Faculty, University of Heidelberg, D-69120 Heidelberg, Germany; setsuko.fujita-becker@bioquant.uni-heidelberg.de (S.F.-B.); rasmus.schroeder@bioquant.uni-heidelberg.de (R.R.S.); 6 Department of Neurology, University Hospital Essen, D-45147 Essen, Germany; mina.borbor@uk-essen.de

**Keywords:** cardiac actin, calcium sensitivity, cardiomyopathies, levosimendan, myosin subfragment 1, myosin binding protein C

## Abstract

Human wild type (wt) cardiac α-actin and its mutants p.A295S or p.R312H and p.E361G correlated with hypertrophic or dilated cardiomyopathy, respectively, were expressed by using the *baculovirus/Sf21* insect cell system. The c-actin variants inhibited DNase I, indicating maintenance of their native state. Electron microscopy showed the formation of normal appearing actin filaments though they showed mutant specific differences in length and straightness correlating with their polymerization rates. TRITC-phalloidin staining showed that p.A295S and p.R312H exhibited reduced and the p.E361G mutant increased lengths of their formed filaments. Decoration of c-actins with cardiac tropomyosin (cTm) and troponin (cTn) conveyed Ca^2+^-sensitivity of the myosin-S1 ATPase stimulation, which was higher for the HCM p.A295S mutant and lower for the DCM p.R312H and p.E361G mutants than for wt c-actin. The lower Ca^2+^-sensitivity of myosin-S1 stimulation by both DCM actin mutants was corrected by the addition of levosimendan. Ca^2+^-dependency of the movement of pyrene-labeled cTm along polymerized c-actin variants decorated with cTn corresponded to the relations observed for the myosin-S1 ATPase stimulation though shifted to lower Ca^2+^-concentrations. The N-terminal C0C2 domain of cardiac myosin-binding protein-C increased the Ca^2+^-sensitivity of the pyrene-cTM movement of bovine, recombinant wt, p.A295S, and p.E361G c-actins, but not of the p.R312H mutant, suggesting decreased affinity to cTm.

## 1. Introduction

Hypertrophic cardiomyopathy (HCM) is frequently characterized by a thickening of the left ventricular wall due to cardiomyocyte hypertrophy accompanied by their disarray. Additional fibrosis leads to increased stiffness of the cardiac muscle and reduced ventricular filling during diastole [1,2].In contrast, dilated cardiomyopathy (DCM) is characterized by enlarged left and/or right ventricles and thinned ventricular walls, leading to a reduced ejection volume during systole [3,4]. HCM has a genetic background in about 90% of all cases with a prevalence of 1:500 in the general population. In contrast, DCM is in only 20% genetically based with a prevalence of 1:2500 [5,6], though there might be many undetected cases within the group of idiopathic DCM [6]. The majority of DCM cases develop as a complication of bacterial or viral infections, adverse lifestyle, alcoholism, drug abuse, or chemotherapy [1,6,7]. 

The hereditary cardiomyopathies (CM) are caused in the vast majority by mutations in genes encoding sarcomeric [1,2] or cytoskeletal proteins [5,8]. About 1400 different mutations causative for HCM or DCM have so far been described. In HCM, the most frequently affected sarcomeric proteins are the ß-myosin heavy chain and the myosin binding protein-C (MyBP-C) [1,6,9]. It has been observed that mutations in genes of cytoskeletal proteins occur more frequently in familial DCM, particularly in components of cell junctions within the intercalated disc [8,10]. In about 20% of the acquired DCM cases, titin gene truncations are observed as de novo mutations [11]. Typically, the CM mutations act in a dominant negative manner and are therefore in most cases single-allelic. Consequently, it is supposed that the mutated protein overrides, in a poisonous manner, the counterpart of the healthy allele [2,12].

**Figure 1:**
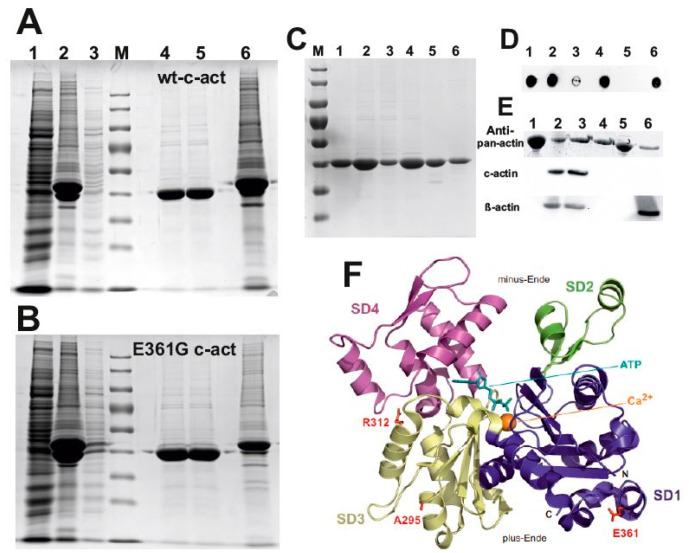
Purification of cardiac actins after expression by the *baculovirus/Sf21* system. (**A**,**B**) SDS-PAGE of purification steps of (**A**) wt c-actin and (**B**) the p.E361G mutant c-actin. Lanes: (1) Sf21 cell homogenate, (2) supernatant after mixture with His-tagged G4-6, and (3) pellet after centrifugation, (M) pre-stained marker proteins, lanes (4,5) purified c-actins after elution of Ni-NTA agarose with EGTA (for details see text), and lane (6) Ni-NTA agarose with His-tagged G4-6 still bound. (**C**) SDS-PAGE of the purified cardiac actin variants used in this study. Lanes: (1) wt recombinant; (2) p.A295S; (3) p.R312K; (4) p.E361G; (5) skeletal muscle actin; and (6) bovine cardiac actin. Actins shown in lanes (5) and (6) were conventionally prepared from acetone powders. (**D**) Dot immunoblots of the actins shown in the same sequence as in (**C**) using the anti-cardiac actin monoclonal antibody. Note that the p.R312K mutant is only weakly and skeletal muscle actin is not stained by anti-c-actin mAB. (**E**) **(Upper row)** SDS-PAGE of the following actins: Lanes: (1) skeletal muscle actin; (2) wt cardiac actin (recombinant); (3) bovine cardiac actin; (4) cytoplasmic β-actin; (5) actin purified from *Acanthameba castellani*; and (6) cytoplasmic β-actin. **(Middle row**) Western blots immunostained with anti-cardiac actin mAB. (**Lower row)** Western blots stained with anti-cytoplasmic β-actin. (**F**) 3D structural model of G-actin derived from the skeletal muscle actin:DNase I complex [25] indicating the positions of the mutated residues of the c-actin mutants investigated. In addition, subdomains (SD) as well as the position of ATP and Ca^2+^ are indicated.

Mutations leading to HCM or DCM have also been identified within the same gene, for instance, in the *ACTC* gene coding for cardiac α-actin (c-α-actin). Mutations of the c-α-actin gene have an incidence of 4–6%, an infrequent finding in patients with familial HCM, and the incidence may be even lower in patients with familial DCM [5,6,12,13,14]. This low incidence contrasts myopathies of skeletal muscle, which are caused to about 30% by mutations of the skeletal muscle actin gene (*ACTA1*) [6,15]. The first mutations of cardiac α-actin that correlated to the cardiomyopathies detected were the p.R312H and p.E361G mutations located in subdomains 4 and 1 of actin (see Figure 1), respectively, causing DCM [16]. The first actin mutant causing HCM detected was the p.A295S mutant located in subdomain 3 (Figure 1A), followed by the p.H90Y and p.R97C mutations both located in subdomain 1 [6,16]. Subsequently, a number of further mutations in c-α-actin have been described that lead to HCM [17]. Presently, 16 mutations with pathological significance have been identified, of which 14 mutations are causative for HCM and two for DCM. Dysfunctions of actin can depend on the type of amino acid exchange, the location of the missense mutation within the molecule, and its effect on the interaction with specific binding partners [6,17]. For example, mutations in subdomain 4 often affect the stability of the actin filament [9,12,13,14,18].

The development of a certain cardiomyopathy phenotype does not proceed by a single pathway. It is complicated by the fact that missense mutations in different sarcomeric proteins can lead to the same disease phenotype. Conversely, point mutations at different locations in the same protein can induce diverse outcomes [7,19]. Therefore, for the development of a particular disease phenotype, even slight alterations of the mutated protein may decisively affect its overall architecture and/or functionality such as its interactions with different binding partners within the sarcomere. Therefore, it appears necessary to analyze in greater detail the properties and functional alterations of each mutated protein. 

Previously, we described the expression of two mutants of c-actin causing HCM (p.Y166C and p.M305L) by the *Sf21/baculovirus* system and their purification in the native state, allowing for their biochemical and cell biological characterization [12]. In the current study, we investigated the functional properties of three further mutants purified after recombinant expression as native and tag-free c-actins, allowing for undisturbed investigations of their properties. The p.A295S mutation located in subdomain 3 has been reported to cause HCM with high penetrance [5] and was shown to perturb the interaction of actin filaments with tropomyosin [20]. The p.R312H and p.E361G mutations are located in subdomains 4 and 1 (Figure 1F), respectively. Both cause DCM [2,10,21] with a benign outcome [13]. 

Here, we compared wild-type (wt) c-α-actin with the p.A295S, p.R312H, and p.E361G mutants in a number of different assays. We also included in this study the p.R312K mutant, which had been suspected to cause DCM (personal communication by Dr. C.-A. Schoenenberger, Basel, Switzerland). So far, however, no report exists indicating that this mutant causes a form of CM. Instead, it appears to represent a polymorphic, not disease related variant, probably because the exchange does not lead to a significant charge alteration. Nevertheless, the data obtained with this variant are given in (Figure 3) because it highlights the fact that the side chain of a missense residue has a considerable effect on its properties.

Our data indicate that after expression in insect *sf21-cells* and purification, the c-α-actin variants were in the native state and able to polymerize albeit with different kinetics and formed normally appearing filaments though of varying lengths. In polymerized form, they stimulated the myosin-subfragment-1 ATPase activity to different extents. After decoration with cardiac tropomyosin (cTm) and troponin complex (cTn), they showed slightly altered Ca^2+^-sensitivities of the stimulation of the myosin-S1 ATPase activity and the azimuthal shift of cTm on their filamentous forms. These alterations might, however, have a larger impact when interacting with other regulatory proteins during cardiomyocyte development or within the intact sarcomere.

## 2. Results

### 2.1. Expression and Purification of the Cardiac α-Actin Variants

Expression of the c-α-actins (wt plus the mutants) was achieved in *Sf21* insect cells by using the *baculovirus* system and purified after affinity binding to His-tagged gelsolin G4-6 as shown for wt and the p:E361G mutant (Figure 1A,B; see also Supplementary Materials and [12]). The isolated proteins showed only one main band after SDS-PAGE (Figure 1C) and were shown to be cardiac α-actin by immunoblotting using an anti-cardiac α-actin antibody (dot blots Figure 1D). The immunoreactivity of purified cardiac-actins, together with skeletal muscle and two cytoplasmic β-actins with isoform specific actin antibodies, is shown in Figure 1E, which shows immunostaining with antibodies directed to all actins (anti-pan-actin, upper row), specific for cardiac actin (middle row), and specific for cytoplasmic β-actin (lower row). It can be seen that the cardiac actins also contained a small amount of cytoplasmic β-actin (previously estimated to be around 10% [12]). The localization of the mutated residues of the isolated c-actin mutants (A295, R312, and E361) on the actin structure is indicated in Figure 1F. 

Since it was suspected that this purification procedure does not discriminate between the expressed cardiac and endogenous insect cell actin, we also performed immunoblots with an anti-β-actin antibody [12,22], assuming that this antibody recognized the endogenous actin of the insect *Sf21*-cells. Indeed, after expression in *Sf21*-cells, the purified cardiac actins possessed β-actin immune-reactivity (Figure 1E). In addition, cardiac α-actin was also conventionally purified from acetone powder obtained from bovine hearts. After SDS-PAGE, its migration behavior and reactivity in western blots against anti-cardiac α-actin was found to be identical to recombinant wt c-α-actin (Figure 1E). Anti-β-actin reactivity was also observed for purified bovine c-α-actin (Figure 1E), probably due to its co-purification from either the fibroblastic cells or cardiomyocytes themselves, where it is localized underneath the plasma membrane and probably within the transition junction adjacent to intercalated discs [23,24].

Initially, we had aimed to isolate the expressed cardiac α-actins using constructs with a N-terminal His-tag. With this procedure, however, only low expression levels were obtained and furthermore, the purified cardiac α-actins did not adopt the fully native configuration as they failed to polymerize and inhibit DNase I. We also generated c-α-actin constructs containing a C-terminal thymosin β4-His-tag [18], however, in our hands, this approach also led to a reduced expression and polymerizability of the c-actins. It was because of these failures that we switched to the previously used procedure using His-tagged gelsolin G4-6 as a means to effectively affinity purify the *Sf21*-cell expressed cardiac actins.

### 2.2. Tests for Native Configuration of the Cardiac Actin Variants

**Figure 2:**
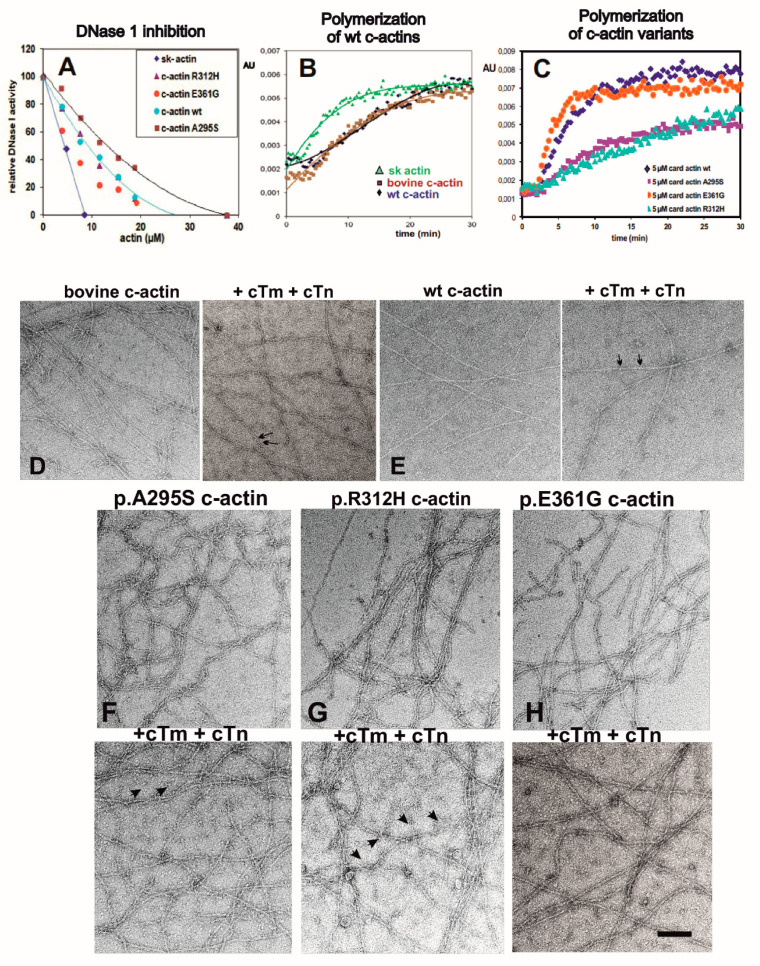
Properties of the purified c-actins indicating native state. (**A**) Inhibition of DNase I activity as measured by the hyperchromicity assay. Human DNase I (0.1 mg/mL = 3.22 μM) was mixed with the amounts of c-actins indicated in the abscissa. DNase activity was determined as detailed [26]. Ordinate gives the relative remaining activity calculated. (**B**) Comparison of the rates of polymerization of purified sk-actin and bovine c-actin purified from acetone powders with recombinant wt c-actin, each at 5 μM. Note the faster polymerization rate of sk-actin and the equal rates of c-actins. Polymerization was initiated after the addition of 2 mM MgCl_2_ at t = 2 min and determined by the pyrene-assay using 0.5 μM pyrene-labeled skeletal muscle actin (see Materials and Methods). (**C**) Polymerization of the cardiac actin variants: recombinant wt-c-actin, p.A295S, p.R312H, and p.E361G (all c-actins at 5 µM; for details see text). (**D**–**H**) Electron microscopy after negative staining of the polymerized c-actins before and after decoration with cTm/cTn: (**D**) bovine and (**E**) wt recombinant c-actin. (**F**) p.A295S, (**G**) p.R312H, and (**H**) p.E361G mutant. Bar in (**H**) corresponds to 100 nm (applicable to all images).

After purification, the native state of the cardiac α-actins was verified by their ability to inhibit DNase I [26]. All of the purified c-α-actin variants inhibited deoxyribonuclease I (DNase I) activity, albeit with different efficiencies. Wt c-α-actin inhibited with a lower efficiency than skeletal muscle actin, needing a nearly three-fold higher concentration for complete DNase I inhibition (Figure 2A). The DCM variants p.R312H/K (for pR312K, see Supplementary Figure S1A) and p.E361G inhibited with a similar or slightly higher efficiency as wt c-α-actin, respectively (Figure 2A). In contrast, the p.A295S mutant showed a clearly lower inhibitory capacity (about 33%) than wt c-α-actin (Figure 2A), though the difference in inhibition efficiency between wt and this mutant c-actin was less prominent than between the wt cardiac and skeletal muscle actin (Figure 2A). These results suggest that the c-α-actin mutants might have attained slightly different conformations because none of the mutations was located within the DNase I-binding loop of subdomain 2 [25]. Native gel electrophoresis used as an additional test for their native conformation confirmed that all c-α-actin variants were able to bind thymosin β4 and gelsolin-segment 1 (G1), as previously described for other c-actin mutants (not shown; [12]). 

### 2.3. Polymerization Behavior of Recombinant c-α-Actins 

The ability of the isolated cardiac actins to polymerize was analyzed by measuring the fluorescence increase of the added pyrenyl-actin [27]. Conventionally purified bovine cardiac actin showed almost identical polymerization behavior expressed as wt c-α-actin (Figure 2B), providing additional confirmation that the expression of the c-actins by the *baculovirus/Sf21* system did not cause an impairment in its functionality. Both cardiac actins polymerized, however, slightly slower than the conventionally purified rabbit skeletal muscle actin (Figure 2B). Clear differences were observed between the mutant c-α-actin variants. The p.E361G DCM mutant showed a slightly higher polymerization rate as wt c-α-actin (Figure 2C). In contrast, the HCM p.A295S and the DCM p.R312H mutant showed significantly reduced rates and extents of polymerization (Figure 2C), as also indicated by the calculation of the half times of their polymerization rates (Table 1. The results for the p.R312K variant are given in Figure S1B.

The critical actin concentrations of polymerization (Cc) were determined separately (see Table 1). The data obtained showed that the Ccs inversely correlated with the polymerization rates (i.e., they were increased for p.A295S and highest for the p.R312H mutant (Table 1), both of which showed lower polymerization rates and extents (Figure 2C)). In contrast, they were lower and did not differ for the other c-actin variants (Table 1). Thus, the observed differences in DNase I inhibition and polymerization efficiency point to missense-specific subtle alterations of their properties, which might be amplified when integrated into sarcomeres of cardiomyocytes. 

### 2.4. Electron Microscopy of Filaments Formed by the c-Actin Variants

Filament formation was further verified by electron microscopy after negative staining (EM) before and after decoration with cardiac tropomyosin (cTm) and troponin complex (cTn) at a molar ratio of 6:1:1 (Figure 2D–H). The data indicated the formation of normally appearing straight actin filaments at this resolution for bovine and wt recombinant c-α-actin (Figure 2D,E), with a very similar length distribution. Filaments of the p.A295S, p.A295S, and p.R312H mutants also formed typical actin filaments, however, frequently exhibited more curved filaments and strand breaks (Figure 2F,G). The length of the p.R312H and p.E361G filaments was very variable, with occasionally long normal appearing but also numerous short filaments (as shown in Figure 2G,H). 

After decoration with cTm/cTn, all mutants appeared to form more regular and longer filaments, as particularly evident for the p.E361G mutant (Figure 2H). Binding of cTn to filaments was detected for bovine and wt c-actin and occasionally for the mutants as regularly spaced small dots (as indicated by arrows in Figure 2D,E), though the decoration of the filaments of the p.A295S and p.R312H with cTm and cTn was not clearly visible, suggesting incomplete decoration. Indeed, thin and short filamentous structures became visible between these actin filaments, probably representing unbound cTm (arrow heads in Figure 2F,G). Release of cTm might have been induced by the negative staining procedure as reported earlier [28]. Even after cTm/cTn decoration, the p.R312H mutant appeared to retain a wavy appearance and fragmentations were still occasionally detectable (Figure 2G).

### 2.5. Properties of p.R312K c-Actin—Probably a Polymorphic Variant 

**Figure 3:**
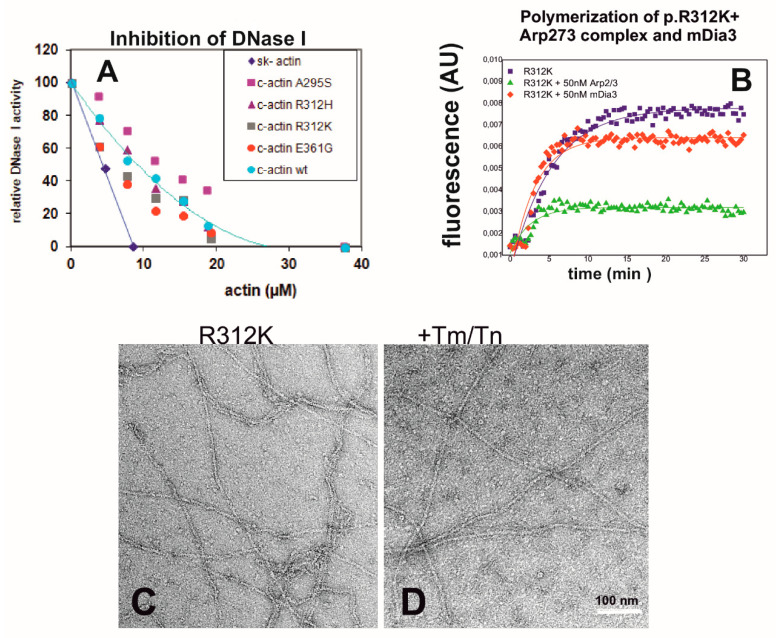
Properties of the purified p.R312K c-actin variant. (**A**) Inhibition of DNase I by the c-actin variants. The inhibition by p.R312K is shown by the trendline. (**B**) Rates of polymerization of the c-actin variants. The rate of 5 µM p.R312K is shown by (♦) and coincides with the rate of wt c-actin. (**C**) Influence of 50 nM Arp2/3 complex and 50 nM mDia3-FH2 of the rate of polymerization of p.R312K. (**C**) EM images of polymerized p.R312K without and (**D**) after decoration with cTm and cTn at 7:1:1 ratio to actin. Bar in (**D**) corresponds to 100 nm.

We also analyzed some properties of the p.R312K variant, which was also expressed by the *baculovirus/Sf9* system and subsequently purified. The R312K c-actin variant possesses a conservative exchange and was suspected to cause DCM (Dr. Cora-Ann Schoeneberger, Basel-University, Basel, Switzerland). Since the p.R312K c-actin variant has not been characterized to date, we investigated some of its biochemical properties. The data shown in Figure 3A indicate that it inhibited DNase 1 equally well as wt-c-actin. Further data showed the rate of polymerization of p.R312K c-actin alone. Its critical concentration of and half time of polymerization were determined to be 0.21 µM and 3.08 min, respectively. In contrast to wt c-actin, neither the Arp2/3 complex nor mDia3-FH2 exerted a stimulatory effect on the rate of polymerization of the p.R312K variant (Figure 3B). In fact, the Arp2/3 complex reduced the extent of polymerization (Figure 3B). The morphological appearance of the p.R312K variant was similar to wt c-actin, as judged by EM after negative staining before and after decoration with cTm and cTn (Figure 3C,D). Furthermore, the p.R312K variant stimulated the myosin-subfragment-1 ATPase activity identically well to wt c-actin, as previously shown [29]. Since no reports exist that demonstrate a pathologic significance of p.R312K or a link to any form of cardiomyopathy, we assumed that it might represent a polymorphic variant, albeit with slightly different properties. Nevertheless, these data demonstrate a profound effect of the side chain at this position when changed to histidine.

### 2.6. Determination of Filament Lengths by Fluorescence Microscopy

**Figure 4:**
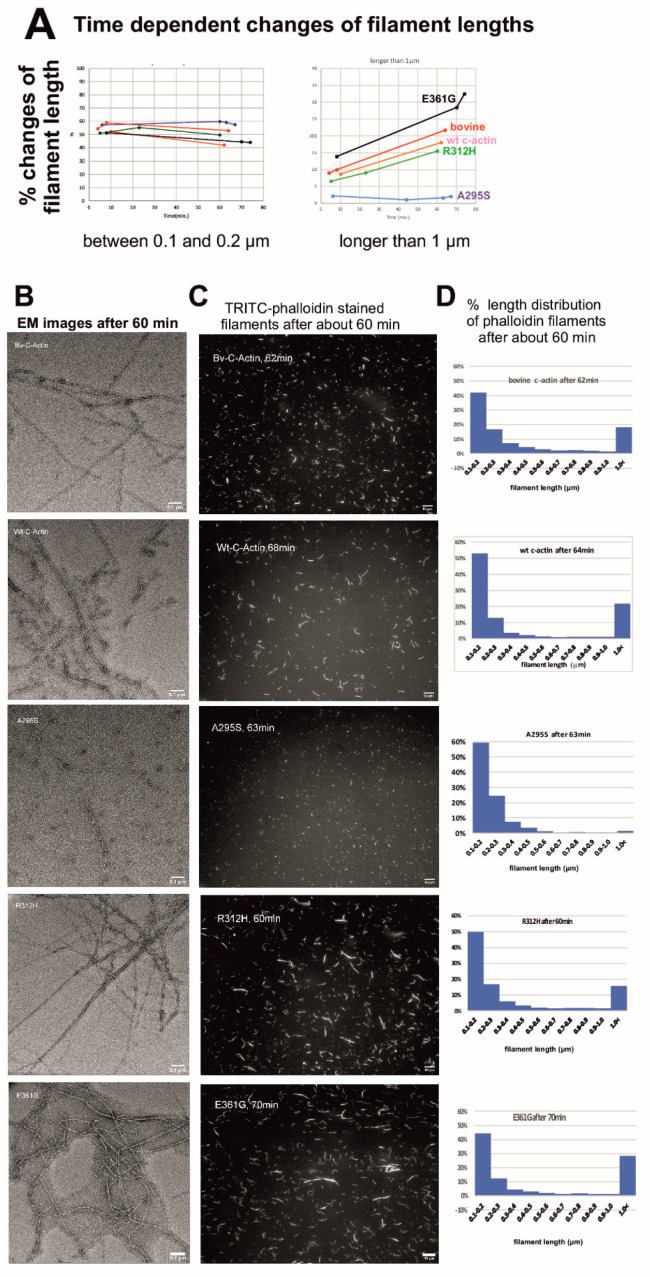
Filament length determination after staining with TRITC-phalloidin. (**A**) Filaments of the cardiac actin variants were labelled with TRITC-phalloidin (see Materials and Methods) and the alterations of their length distribution were followed over time for filaments of a length between 0.1 and 0.2 µm and over 1 µm. Note that the number of filaments between 0.1 and 0.2 µm remained almost constant, whereas the number of filaments over 1 µm increased, except for the p.A295S mutant. The largest increase was noted for the p.E361G mutant. (**B**) The appearance of the phalloidin labelled filaments was visualized by EM after about 60 min and (**C**) by fluorescence microscopy. (**D**) Shows the percental length distributions after about 60 min as determined by ImageJ Ridge Detection (see Materials and Methods). Bars in (**B**,**C**) correspond to 100 nm and 10 µm, respectively.

Since filament lengths cannot be reliably determined by EM, we analyzed the length distribution of the c-actin filaments by using fluorescence microscopy after labelling with TRITC-phalloidin (see Materials and Methods). The time dependent changes in the filament length distribution shown in Figure 4A demonstrate a slight percental decrease in short (length between 0.1 and 0.2 µm) and a percental increase in long filaments (longer than 1 µm) after about 60 min of incubation with TRITC-phalloidin added at a molar ratio of one to two actin subunits. Normal filament formation was verified by EM (Figure 4B). The real length distribution became visible by fluorescence microscopy (Figure 4C) and was subsequently quantified from microscope images using ImageJ Ridge Detection (Figure 4D). The data clearly showed that after 60 min, the number of filaments formed by the pA295S mutant did not increase and only slightly by the p.R312H mutant: the percental number of p.R312H filaments longer than 1 µm increased only from 7% to 15% (Figure 4A,D). In contrast, the pE361G mutant formed considerably longer filaments than wt c-actin (Figure 4A,D). These relations did not change considerably after incubation times longer than 60 min, except for the p.E361G mutant, which then formed slightly longer filaments. In addition, the fluorescence microscopy showed that the p.R312H and p.E361G filaments were often bundled, a behavior that was not observed for bovine or wt c-actin (Figure 4C).

### 2.7. Stimulation of the Myosin-S1 ATPase Activity by Filamentous c-Actins

**Figure 5:**
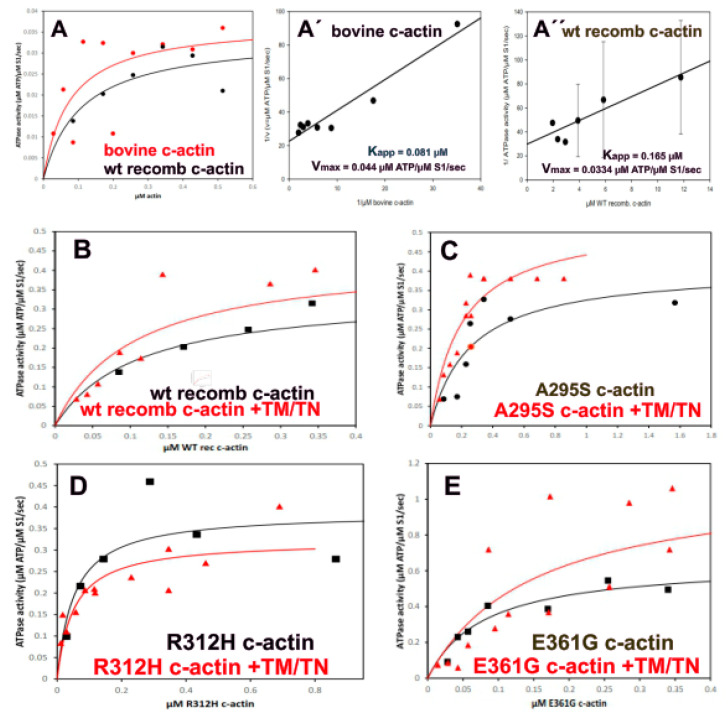
(**A**) Gives the dependence of the myosin-S1 ATPase stimulaztion on the concentration of bovine and recombinant wt c-actin. (**A′**,**A″**) give examples of the double reciprocal evaluation of the myosin-S1 ATPase stimulation by bovine and recombinant wt c-actin, respectively. The intercept of the linear slopes with Y-axis gives 1/V_max_ and the X-axis gives minus 1/K_M_, which like a Michaelis-Menten constant can be taken as an apparent binding constant. Since both values are not determined kinetically, they are named “apparent”. The results of an identical evaluation of the c-actin mutants are given in Table 2. (**B**–**E**) Stimulation of the myosin-S1 ATPase by increasing concentrations of the c-actin variants in the absence and presence of cTm/cTn. The ability of filamentous c-actin variants was determined by an enzyme-linked optical assay [30]. The c-actins were polymerized by 2 mM MgCl_2_ plus 50 mM KCl. The ATPase activity was determined for 1 µM rabbit skeletal muscle myosin-S1 prepared according to [31] in 5 mM HEPES-HCl, pH 7.4; 0.1 mM CaCl_2_, 2mM MgCl_2_, 50 mM KCl NADH phosphoenolpyruvate plus lactate dehydrogenase and pyruvate kinase. The time dependent decrease in optical density at 340 nm was determined with a Beckman DU640 photometer.

In striated muscle cells, myosin heads protruding from the thick filaments interact with actin filaments during force production. The energy for this process is provided by the actin stimulated ATP-hydrolysis by myosin motor domains. First, we determined the concentration dependence of the stimulation of the myosin-subfragment-1 (myosin-S1) ATPase activity by the F-c-actin variants at high Ca^2+^-concentration (at pCa^2+^ = 4.3) before and after decoration with cTm/cTn (at a molar ratio of 6:1:1), in order to compare the standard kinetic parameters. The data (Figure 5) showed that the polymerized c-actins stimulated the myosin-S1 ATPase activity before and after decoration with cTm and cTn. Decoration with cTm/cTn, however, increased their stimulatory activity except for the p.R312H mutant. In addition, we observed that wt recombinant and bovine c-actin possessed similar stimulatory activity in the presence or absence of cTm/cTn, as shown by the double reciprocal plots (Figure 5A′,A″), which allowed us to determine the apparent K_M_ (apparent binding affinity of myosin-S1 to the particular F-c-actin) and Vmax values (the maximal stimulation of the myosin-S1 ATPase activity). A compilation of these parameters for the cardiac actins is given in Table 2. The reciprocal analyses showed that the K_M_ values varied considerably. In the absence of cTm/cTn, the Vmax values were rather similar for all c-actins, but indicated a decrease in the apparent affinity (K_M_) of the mutant c-actins to myosin-S1 after decoration with cTm/cTn, except for the p.R312H mutant (Table 2). After decoration with cTm/cTn, the Vmax values increased, except for the p.R312H mutant. The increase in Vmax was highest for the p.A295S mutant, suggesting the stabilization of its F-actin formation. 

### 2.8. Ca^2+^-Dependence of the Myosin-S1 ATPase Stimulation by c-α-Actin Variants 

**Figure 6:**
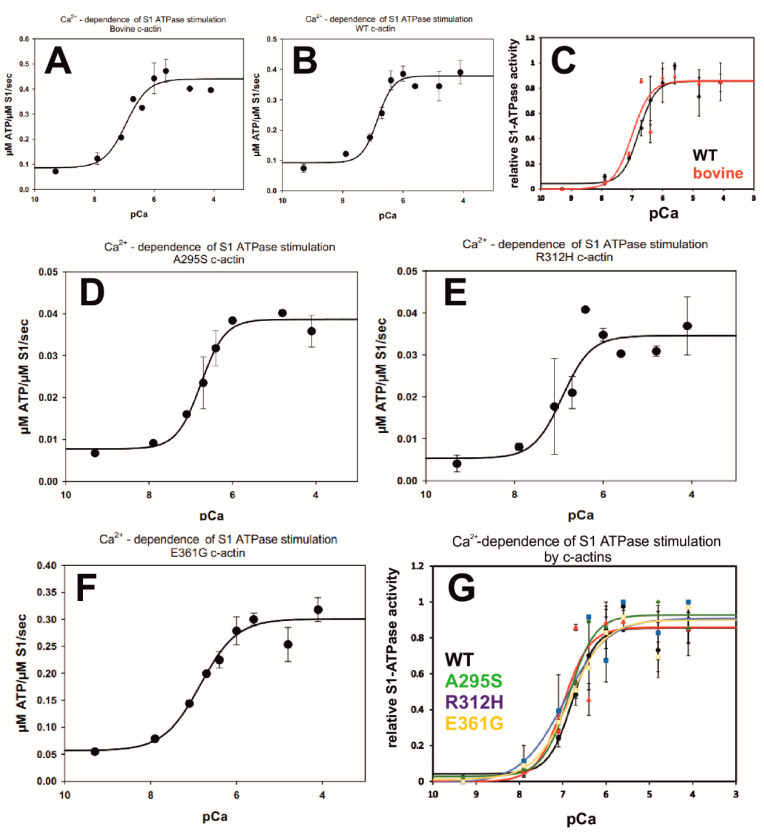
Ca^2+^-concentration dependence of the stimulation of the myosin-S1 ATPase by c-actins decorated with cTm and cTn at a 6:1:1 molar ratio. (**A**) Bovin c-actin, (**B**) WT recombinant actin, (**C**) Bovine vs. WT recombinant actin, (**D**) A295S c-actin, (**E**) R312H c-actin, (**F**) E361G c-actin, (**G**) mutant actins vs. WT recombinant actin.

The interaction of myosin motor domains with F-actin decorated with cTm/cTn is regulated by an increase in cytosolic Ca^2+^-ion concentration released from the sarcoplasmic reticulum. Therefore, we determined the Ca^2+^-concentration dependence of the myosin-S1 ATPase stimulation after decoration of the filamentous c-actins with cTm/cTn (at 7:1:1 ratio). The c-actins were decorated by native cTm or pyrene-labelled cTm (see below) without any difference in their ability to convey Ca^2+^-dependent stimulation of the myosin-S1 ATPase. The absolute ATPase rates (shown in Figure 6A,B,D–F) indicate a rather similar extent of stimulation, although the maximal ATPase stimulation was highest for bovine c-α-actin and p.A295S (Table 3). Superposition of the normalized rates indicated that bovine and recombinant wt c-α-actin stimulated the myosin-S1 ATPase with a similar Ca^2+^-dependence (Figure 6C and Table 3). The superposition of the Ca^2+^-dependences of the stimulation of the myosin-S1 ATPase by wt and the three c-α-actin mutants (Figure 6G) indicated that the p.A295S mutant showed a slightly higher (especially at low Ca^2+^-concentration) and the two DCM mutants (p.R312H and p.E361G) a similar or slightly lower Ca^2+^-sensitivity in comparison to wt c-α-actin (Figure 6G).

The pCa_50_ values and differences in Hill coefficients are compiled in Table 2. These data show that after cTm/cTn decoration, in comparison to wt c-actin and the two DCM mutants, the p.A295S mutant possessed a higher Ca^2+^-sensitivity. The Hill coefficients (Table 3) appeared to be rather similar for wt and the p.A295S mutant, however, the DCM mutants p.R312H and p.E361G and bovine c-actin showed lower Hill coefficients (i.e., the ability for cooperative propagation of myosin-S1 binding along the actin filament). The reduced Hill coefficient of the DCM mutants might be due to the fact that the p.R312H and p.E361G mutations are located within the cTm and myosin head binding regions, respectively (see also Discussion). 

Student t-distribution tests and R-square (R^2^) analysis indicated that the measurements were significant for each special condition but not of high significance when comparing different mutants with each other or under different conditions (varying compositions of actin-binding proteins, see also Table 3).

Skeletal muscle myosin-S1 ATPase activity was determined by the enzyme-linked assay. ATPase stimulation by (A) recombinant wt c-actin, (B) bovine c-actin, and (C) comparison of (A) and (B). ATPase stimulation by (D) p.A295S, (E) p.R312H, and (F) p.E361G. (G) Comparison of the mutant forms of c-actin. Final F-c-actin concentrations in the assay were 1.5 μM and of rabbit skeletal muscle myosin-S1 0.5 μM. ATPase activity (ordinate) is given as μM ATP hydrolyzed/sec/μM myosin-S1 in (A; B; D–F) and normalized in (C and G) setting the minimal ATPase activity (generally at pCa 9.3) as zero and the maximal at 1 (generally at pCa 4.8). Abscissa gives pCa-values (−log molar Ca^2+^-concentration). All measurements were performed in triplicate. 

### 2.9. Levosimendan Affects the Ca^2+^-Dependence of Myosin-S1 ATPase Stimulation by the DCM c-Actin Mutants 

**Figure 7:**
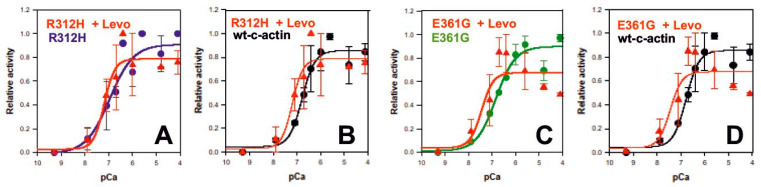
Influence of levosimendan on the stimulation of myosin-S1 by the DCM c-actin mutants. The Ca^2+^-concentration dependence of myosin-S1 ATPase stimulation by the DCM mutants p.R312H and p.E361G were determined in the presence and absence of 20 µM levosimendan by the enzyme-linked optical assay using 1 µM skeletal muscle myosin-S1 and 1.5 µM of the polymerized c-actin mutants decorated with cTm/cTn at a molar ratio to the polymerized c-actin mutants of 6:1:1. (**A**): p.R312H ± Levo; (**B**): p.R312H ± Levo and wt c-actin; (**C**): p.E361G ± Levo; (**D**): p.E361G ± Levo and wt c-actin. Data are given as mean values ± SEM. Abscissa gives pCa-values (-log molar Ca^2+^-concentration). All measurements were performed in triplicate.

Next, we tested whether the cTn-binding Ca^2+^-sensitizer levosimendan was able to shift the Ca^2+^-dependent myosin-S1 ATPase stimulation by the DCM c-actin mutants p.R312H and p.E361G to lower Ca^2+^-concentrations. Indeed, in the presence of 20 µM levosimendan, we obtained a clear left shift of the pCa curves of myosin-S1 stimulation by these mutants (Figure 7). In the presence of levosimendan, the p.R312H mutant attained a pCa_50_ of 7.19 ± 0.20 with a Hill coefficient of 1.9 ± 1.92 and the p.E361G mutant a pCa_50_ of 7.44 ± 0.34 with a Hill coefficient of 1.75 ± 1.36, whereas in the absence of levosimendan, the corresponding values for the pR312H or p.E361G mutant were a pCa_50_ of 6.923 and Hill coefficient of 1.342 or a pCa_50_ of 6.844 and Hill coefficient of 1.021, respectively (the results are summarized in Table 4). These data suggest that the left shift of the Ca^2+^-sensitivity might be due to higher Hill coefficients induced by levosimendan (i.e., that this drug increased the cooperativity along the mutant actin filaments, probably by increasing the strength of Ca^2+^-binding to TnC and subsequently inducing the cTm-shift to the open position at lower Ca^2+^-concentrations). 

### 2.10. Ca^2+^-Dependence of Tropomyosin Movement on c-α-Actin Variants

Binding of one Ca^2+^-ion to the cardiac troponin subunit TnC induces a conformational alteration of the cTn complex, leading to reduced binding of TnI to actin and an azimuthal movement of Tm along the actin filament and the exposure of binding sites for myosin heads [30]. Functionally, three different Tm positions have been identified: (i) in relaxed muscle the blocked (B-)state that inhibits myosin head attachment; (ii) upon cytosolic Ca^2+^-ion increase and binding to TnC, tropomyosin moves to the closed (C-)state position that allows binding of myosin heads with low affinity; and (iii) finally induced by further myosin head binding the open (M-)state position, allowing their binding with high affinity [28,32,33,34,35]. The extent of Tm movement is largest (azimuthal angle of about 20 degrees) when going from the B- to the C-state, whereas the transition from C- to M-state requires only a smaller azimuthal movement (angle change about 5 degrees). Due to a lower energy barrier, the transition from the C- to M-state occurs much more readily in cardiac than skeletal muscle [36]. 

In cardiac muscle, most of the tropomyosin is a homodimer composed of two α-chains. Labeling of cTm with pyrenyl-maleinimide occurs at Cys190 of both chains and produces a fluorescent eximer [37] that has been used to monitor its binding to F-actin [38]. Furthermore, it has been shown that in fully reconstituted thin filaments, the fluorescence change is a direct measure of the myosin-S1 induced Tm movement [38,39]. We used this assay to analyze whether the observed Ca^2+^-sensitivities of the myosin-S1 ATPase stimulation are paralleled by a similar behavior of the cTm movement (i.e., a fluorescence increase in pyrene-cTm). For this aim, polymerized c-actin variants were decorated with pyrene-labeled cTm and cTn at a 6:1:1 molar ratio. The Ca^2+^-dependent changes in pyrene-cTm fluorescence were followed by using a microtiter assay read by a microplate-reader (see Materials and Methods).

**Figure 8:**
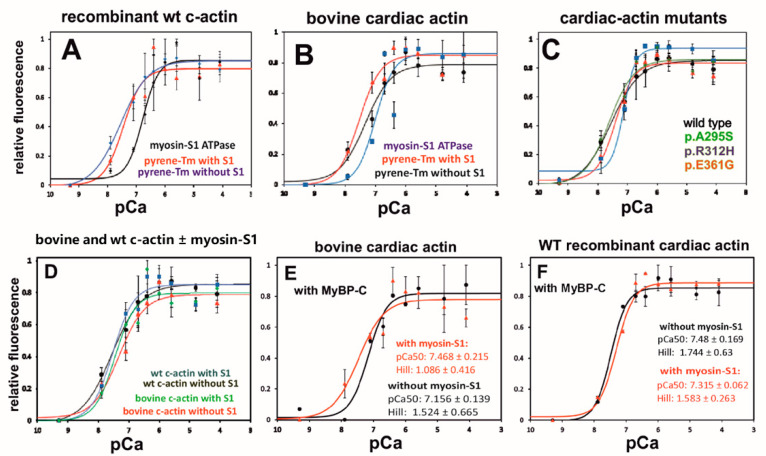
Comparison of the Ca^2+^-concentration dependence of the fluorescence increase (movement) in pyrene-labeled cTm on bovine and recombinant c-actin variants.

The data obtained generally indicated a higher Ca^2+^-sensitivity for the pyrene-cTm movement than that determined by the myosin-S1 ATPase stimulation (for an explanation see Discussion). Figure 8A shows the higher Ca^2+^-sensitivity of pyrene-cTm movement bound to recombinant wt c-actin in the absence and presence of myosin-S1 than for the myosin-S1 ATPase stimulation. Figure 8B shows the same comparison for bovine c-actin. For both c-actins, half maximal fluorescence change was achieved at about 5 × 10^−8^ M versus 10^−7^ M CaCl_2_ for half maximal ATPase stimulation (Table 3 and Table 5). Of note, the addition of myosin-S1 further increased the Ca^2+^-sensitivity of the pyrene-cTm fluorescence increase (i.e., cTm movement) when bound to bovine but not when attached to recombinant wt c-actin (Figure 8B; Table 5; see also the Discussion). Similarly, the Ca^2+^-sensitivity of pyrene-cTm movement along the c-actin mutants was also higher than that of their stimulation of the myosin-S1 ATPase, but was not further increased after myosin-S1 addition (Figure 8C; Table 5). Interestingly, the Ca^2+^-sensitivity of pyrene-cTm movement on bovine c-actin in the presence of myosin-S1 coincided with that of wt c-actin (±myosin-S1), suggesting that cTm bound to wt c-actin had attained the M-state irrespective of the presence of myosin-S1 (Figure 8D). 

The p.A295S mutant showed a slightly lower pCa_50_ than recombinant wt c-actin, but a higher Hill coefficient (Figure 8C, Table 5), leading to a higher Ca^2+^-sensitivity at Ca^2+^-concentrations above its pCa_50_. In contrast, in the presence of myosin-S1, the p.R312H and p.E361G mutants showed a clearly reduced Ca^2+^-sensitivity when compared to wt and the p.A295S mutant (Figure 8C; Table 5).

In addition, this assay allowed for the determination of the Ca^2+^-sensitivities of the cTm-movement in the absence of myosin-S1. The data compiled in Table 5 indicate that for the p.A295S mutant, a higher Ca^2+^-sensitivity than wt and p.E361G c-actin. The Hill coefficients determined appeared rather similar, except for the p.R312H mutant, which showed the lowest Ca^2+^-sensitivity but the highest Hill coefficient (of 3.9), suggesting that this mutation allowed, only at higher Ca^2+^-concentration, a sudden transition from B- to C-state, probably due to a weakened cTm binding in the C-state. This effect is probably due to a charge alteration of the so-called A-triad—a cluster of three positive residues on subdomain 3 of actin forming the binding site of Tm during the C-state [7,20]. In the presence of myosin-S1, however, the Hill coefficient of p.R312H c-actin was reduced to 1.28, probably because myosin-S1 simultaneously binds actin and tropomyosin [33] (and see Discussion).

Pyrene-labeled cTm was used to determine its movement along filamentous bovine and recombinant wt c-actin (for details see text). The c-actins (final concentration in microtiter plate 0.4 μM) were decorated with pyrene-labeled cTm and cTn at a molar ratio of 6:1:1 (see Materials and Methods) and the Ca^2+^-dependence of the fluorescence increase in the pyrene-labeled cTm corresponding to Tm-movement was determined using a Tecan Elisa-reader, as detailed in the Materials and Methods [9]. (A) Comparison of the Ca^2+^-dependence of the stimulation of the myosin-S1 ATPase by recombinant wt c-actin with that of the increase in fluorescence of pyrene-cTm in the absence and presence of myosin-S1. Note lower Ca^2+^-sensitivity of the myosin-S1 ATPase stimulation. (B) Similar comparison for bovine c-actin and wt c-actin decorated with pyrene-cTm and cTn in the absence and the presence of N-cMyBP-C. (C, D) Identical experiments to (A) and (B) using the recombinant c-actins. (E, F) Identical experiment to (C) and (D) using the recombinant c-actins in the additional presence of myosin-S1. The ordinates give the fluorescence intensities (F) normalized to Fmax = 1, Fmin = 0. The abscissa gives the pCa (for compilation of data see also Table 4). The color coding for the c-actins is given in Figure 5.

The c-actin variants were decorated with pyrene-labelled cTm at a ratio of 6:1 and the dependence of the increase in pyrene-fluorescence on Ca^2+^-concentration was determined (see Materials and Methods) in the absence or additional presence of myosin-S1 alone and/or plus N-cMyBP-C. Hill coefficients, pCa_50_-values, and the R^2^-coefficient were calculated by Sigma Plot Systat software (Erkrath, Germany). n gives the number of experiments. Variations are given as SEM.

### 2.11. Effect of the N-Terminal C0-C2 Segment of Myosin-Binding Protein-C on the Ca^2+^-Dependency of Pyrene-cTm Movement

Within the intact sarcomere, a number of associated proteins modulate the activity of the contractile machinery such as the myosin-binding protein-C, which extends from the thick to the thin filaments within the so-called C-region of the A-band (overlap region) [40]. The cardiac specific isoform has been shown to activate acto–myosin interaction at low Ca^2+^-concentration [9,41,42]. Skeletal muscle MyBP-C is composed of seven immunoglobulin (IgG) and three fibronectin (FN) domains. Cardiac MyBP-C possesses an additional cardiac specific C0-domain at its N-terminus and between the C1 and C2 domain, the so-called M-domain, which in native muscle is phosphorylated in response to external signals [41]. In our experiments, however, we used an N-terminal part composed of the C0–C2 domains, which was recombinantly expressed and therefore non-phosphorylated N-cMyPB-C. This C0-C2 N-terminal part of cMyBP-C is able to bind actin and the head region of myosin [43,44]. Binding to actin of N-cMyBP-C occurs mainly through the C1-domain and is supported by the C0-domain, which has been reported to also bind to actin or to the myosin head or its regulatory light chain [42].

It is generally accepted that MyBP-C broadens the Ca^2+^-dependency of muscle activation, most probably due to the ability of the C1-domain to also bind to cTm and to shift its position toward the C-state, thereby increasing the probability of myosin-heads to bind to actin at low Ca^2+^-concentrations [8,45]. Of note, mutations of cMyBP-C are one of the most frequent causes of cardiomyopathies [44].

Because our data indicated alterations of the interaction of the mutant c-actins with cTm, we analyzed whether N-cMyPB-C altered the Ca^2+^-sensitivity of pyrene-cTm movement bound to the c-actin variants. The measurements were performed under four different conditions: in the absence and presence of N-cMyBP-C and of cardiac myosin-S1. The F-c-actins were decorated with pyrene-cTm, cTn, N-cMyBP-C, and myosin-S1 at a six-fold lower molar concentration to actin subunits. A broadening of the Ca^2+^-sensitivity of the pyrene-cTm movement will be reflected by a decrease in the Hill coefficient of the sigmoidal fluorescence increase. The data obtained are compiled in Figure 8E,F (and Table 5) and indicate that N-cMyBP-C elicited c-actin variant specific differences of the pyrene-cTm fluorescence increase. When added to bovine F-c-actin, a decrease in the pCa_50_ and the Hill coefficient was observed in the presence of myosin-S1. For recombinant wt F-c-actin, we detected, only in the presence of myosin-S1, a decrease in the pCa_50_, but no change in the Hill coefficient. In contrast, the p.A295S and p.E361G mutants showed a lowering of the Hill coefficient in the absence and presence of myosin-S1, the p.R312H mutant only in its absence. N-cMyBP-C, however, showed no significant effects on the pCa_50_-values of the mutants.

## 3. Discussion

Hypertrophic and dilated cardiomyopathies are the most frequent genetic diseases of the heart and caused to a high percentage by point mutations of genes encoding sarcomeric proteins. Here, we analyzed the biochemical and physiological properties of three mutations of cardiac α-actin correlated to cardiomyopathies. For this study, wild type cardiac α-actin, the p.A295S mutant causing HCM, and the p.R312H and p.E361G mutants correlated to DCM were expressed using the *Sf21/baculovirus* system, isolated in native state, and analyzed. It was hoped that the analysis of actin mutants, causative for either of these two CM forms, might reveal distinctly different properties that could be specifically attributed to a particular CM disease phenotype. 

After isolation, the cardiac α-actins were pure as judged by SDS-PAGE, but appeared to contain about 10% cytoplasmic ß-actin as revealed by western blotting with an anti-ß-actin specific antibody. Nevertheless, we are confident that the properties and activities of the purified α-actins are attributable to the cardiac actins, since the observed deviations from the wild type behavior can only be due to alterations of the properties of the cardiac isoforms.

Point mutations of proteins can lead to loss or alterations of the native state, restricting their functionality. The purified c-α-actins appeared to be in native state as verified by their ability to inhibit DNase I activity though with different efficiencies. These data may suggest slight structural alterations, since none of the point mutations were localized in subdomain 2, the binding region of DNase I [25]. Bovine, wt, and the p.E361G mutant c-α-actin showed similar polymerization behavior, suggesting identical functionality of the interaction sites involved in filament formation. The p.A295S and p.R312H mutants polymerized slower and less efficiently (i.e., they formed shorter filaments than the other variants). Indeed, their critical concentrations of polymerization (Cc) were higher and half-times of polymerization were longer, suggesting slight alterations in their mode of self-interactions. Electron microscopy after negative staining indicated that all c-actin variants formed typical actin filaments. The filaments formed by bovine, wt c-actin, and the p.A295S mutant showed a similar appearance. Differences were, however, observed concerning their filament straightness and the presence of breaks: the DCM p.R312H and p.E361G mutants showed frequently bent filaments and fragmentations. 

The length distribution was analyzed by fluorescence microscopy after labeling with TRITC-phalloidin because such measurements cannot reliably be performed by EM due to its much higher magnification. The data showed decreased mean lengths for the p.R312H mutant, particularly for F-p.A295S, and considerably higher percentage of long filaments for F-p.E361G. The data concerning the length of filaments of the p.A295S mutant are in contrast to an earlier report suggesting normal or even longer filament length after expression in *Drosophila* cardiac tube or flight muscles [20]. This result, however, might have been due to the co-expression with endogenous actin after their transfection into neonatal cardiomyocytes [29,46].

In muscle, the function of F-actin is to stimulate the myosin ATPase activity. Myosin-S1 of skeletal or cardiac muscle represents the globular N-terminal head domain of myosin that contains both the actin binding and the separate ATPase site. Our data show that every c-α-actin isoform was able to stimulate the myosin-S1 ATPase in a concentration dependent manner (see Figure 5). Decoration with cTm/cTn led to an increase in the myosin-S1 ATPase stimulatory activity of wt, p.A295S and p.E361G c-α-actin, probably due to stabilization of their filament structure, except for the p.R312H mutant, whose myosin-S1 stimulatory activity decreased after cTm/Tn decoration (Figure 5). The obtained kinetic parameters from these measurements are summarized in Table 2, which demonstrated a higher myosin-S1 ATPase stimulatory capacity (V_max_) of p.A295S before and after decoration with cTm/cTn than the wt and the DCM c-α-actin variants. Surprisingly, the p.R312H mutant showed the lowest apparent K_M_-value, suggesting a considerably stronger affinity of myosin-S1 than the other c-actins. Furthermore, decoration of the p.R312H mutant with cTm/cTn led to a further decrease in the apparent K_M_-value, probably causing the inhibitory effect of cTm/cTn on its myosin-S1 ATPase stimulatory activity (Table 2). These data demonstrate that point mutations of a particular c-actin residue can lead to significant alterations of its interaction with a particular binding partner, for instance, myosin heads.

Decoration of all c-α-actin variants with cTm/cTn conferred Ca^2+^-sensitivity of their myosin-S1 ATPase stimulatory activity that underlined their functionality. However, differences in the pCa_50_-values were observed for their myosin-S1 ATPase stimulatory activity. The p.A295S HCM mutant showed an at least equal or slightly higher Ca^2+^-sensitivity than wt c-α-actin, however, the pCa_50_-values of both were clearly higher than that of the two DCM variants (see Table 3 and Table 5). The data are in line with the assumption that HCM is correlated with a high and DCM with decreased Ca^2+^-sensitivity, leading to a higher or lower contractile activity, respectively [47]. 

The p.A295S mutant was the first c-actin mutation identified through clinical research and shown to cause HCM [16]. The A295 residue is close to the Tm binding region and in Rigor state close to loop 4 of myosin [33,48], but previous studies have suggested that the p.A295S mutant exhibits little differences to wt-c-actin [49,50]. A recent study, however, described the expression of A295S c-actin in the cardiac tubes and indirect flight muscle of Drosophila melanogaster [20]. This in vivo system demonstrated increased Ca^2+^-sensitivity and reduced speed of relaxation that led to hypercontractile muscles when expressing the A285S mutant in insect cardiac tubes [20]. Our study reached a similar conclusion based on biochemical assays, though it appears that the mutational effect is more clearly evident in an in vivo system, possibly due to further interactions or structural organization that cannot be completely reconstituted in biochemical assays.

The differences in the Ca^2+^-regulatory activities of the mutant c-actins were confirmed by measurements of the Ca^2+^-sensitivity of cTm movement by determining the Ca^2+^-dependent fluorescence increase in pyrene-labeled cTm. The data indicated that the pCa_50_ differences between the c-α-actins were principally maintained when using this procedure, though the fluorescence signals were generally shifted to lower Ca^2+^-ion concentrations (Figure 8 and Table 5). The reasons for the discrepancy between the two different procedures (ATPase versus pyrene-cTm) may be due to the reported weakened interaction of the cTnT subunit to the pyrenyl-modified region (Cys190) of cTm, leading to an alteration of the Ca^2+^-dependent binding of the cTnI subunit to actin [51]. Tm binding along F-actin is purely electrostatic and of low affinity [5,36], and the introduction of two hydrophobic pyrene-moieties may have further reduced its affinity to F-actin, resulting in a shift in the Ca^2+^-dependency to lower Ca^2+^-concentrations.

Nevertheless, this procedure also allowed for the determination of the Ca^2+^-dependency of the movement of pyrene-cTm in the absence of myosin-S1, presumably corresponding to its movement from the B- to C-state. For bovine c-actin, we observed a Ca^2+^-dependent increase in pyrene-fluorescence in the absence of myosin-S1, possibly corresponding to the cTm movement from the B- to C-state, which was further left-shifted after the addition of myosin-S1. This second shift to lower Ca^2+^-concentrations by myosin-S1 most likely corresponds to Tm-movement from the C- to M-state. This two-step effect was only observed for bovine c-actin and the p.R312H mutant in the presence of N-cMyBP-C (Table 4), but not for the other recombinant c-actin variants, which showed, irrespective of the presence of myosin-S1, the Ca^2+^-dependency of the fluorescence increase in bovine c-actin plus myosin-S1. Presumably, these c-actin variants responded to the Ca^2+^-concentration increase with an immediate complete shift of pyrene-cTm to the presumed M-state, as proposed earlier [36]. Though the reason for this difference is presently unclear, it might, however, be caused by a higher content of skeletal muscle α-actin in the conventionally prepared bovine c-actin. Indeed, it is known that cardiac muscle sarcomeres contain up to about 20% skeletal α-actin, which shows a higher energy barrier of the Tm transition from the C- to M-state [35]. 

We also determined the Ca^2+^-sensitivity of the c-actin variants decorated with pyrene-cTm/cTin in the presence of the N-terminal fragment C0C2 of cMyBP-C. MyBP-C was first discovered in skeletal muscle bound to the thick filaments, where its occurrence is restricted to the overlap region of thick and thin filaments [7]. MyBP-C is also able to attach to actin and titin, thereby connecting thin and thick filaments. Meanwhile, three isoforms of MyBP-C were identified: skeletal, smooth, and cardiac muscle specific isoforms [40,41]. Cardiac MyBP-C is composed of eight immunoglobin-like and three fibronectin-like domains, which are numbered from C0 (N-terminus) to C10 [41]. C0 and C1 are linked by a flexible proline-alanine rich linker and between C1 and C2 is placed the M-domain, which is phosphorylated by a number of signaling protein kinases [40,41]. In our experiments, we used the N-terminal and actin-binding fragment C0–C2 of cardiac MyBP-C, which has been shown to broaden the Ca^2+^-response (decrease of the Hill coefficient) and thereby to increase the Ca^2+^-sensitivity of native thin filaments at low Ca^2+^-concentrations [41]. Due to its recombinant expression, the C0C2-fragment used was non-phosphorylated and probably unable to bind myosin-S1. 

Our data showed that N-cMyBP-C slightly shifted the pCa_50_ of bovine and wt c-α-actin to lower Ca^2+^-concentrations (increase the Ca^2+^-sensitivity), whereas its effect on the mutant c-α-actins varied from none to a slight increase in Ca^2+^-sensitivity of p.R312H and p.E361G in the absence of myosin-S1 (Table 3). These different responses might be due to alterations of the interaction of N-cMyBP-C with the mutated actins. Indeed, previous data have shown that the affinity of the C0C2 fragment is reduced to mutated c-actins, especially to the p.E361G mutant [8], because the E361G mutation is located in subdomain 1 and is possibly close to the presumed binding region of N-cMyBP-C [41]. 

## 4. Materials and Methods

### 4.1. Antibodies and Reagents

Monoclonal mouse anti-skeletal α-actin (clone AC-1-20.4), anti-α-actin (clone AC15), and rabbit anti-actin pan (clone C11) antibodies were obtained from Sigma-Aldrich (Munich, Germany). Mouse anti-cardiac α-actin monoclonal antibody was purchased from Progen Biotechnik GmbH (Heidelberg, Germany). Pyrene-maleinimide was obtained from Sigma Aldrich (Munich, Germany). All other reagents were of analytical grade.

### 4.2. Clones

The pcDNA3.1/NT-GFP-TOPO^®^-WT-α-cardiac actin and the mutants p.A295S, p.R312K, and p.E361G were donated from Dr. Cora-Ann Schoenenberger (University Basel, Switzerland). The p.R312H mutant was generated by site-directed mutagenesis from the p.R312K variant. The c-α-actin-containing plasmids served as templates for cloning the c-α-actin variants into the p3xHA-C1 plasmid. The p3xHA-C1 plasmid was a kind gift from Dr. T. Engel (Leibniz-Institut für Arteriosklerosis, Münster University, Germany), who deleted cDNA of EGFP from the pEGFP-C1 plasmid (Clontech, Mountain View, California, USA) and instead cloned into this plasmid the cDNA of a three times repeated hemagglutinin-tag (HA). The primers used for amplifying the actin cDNAs were as follows: 5′-GTTATGTGTGACGACGAGGAGACC-3′ and 5′-ATTGCCCTTTTAGAAGCATTTGCG-3′. PCR inserts were cloned into p3xHA-C1 using *Xba*I and *Xho*I sites.

The deletion construct of human gelsolin G4-6 was kindly supplied by Dr. A.G. Weeds (MRC-LMB, Cambridge, UK) and subcloned from shuttle vector pKN172 into the cold-shock expression plasmid pCOLD II (Takara Bio Inc., Kusatsu, Japan) using the restriction sites for *Bam*HI and *Hind*III enzymes obtained from Fermentas (Vilnius, Lithuania). The pCOLD II plasmid provides a His-Tag sequence for affinity chromatography, which was fused to the N-terminus of G4-6 and subsequently used to affinity purify the c-α-actins [12,46].

### 4.3. Protein Expression and Purification

Rabbit skeletal muscle and bovine c-α-actin were prepared from dried acetone powder and human cardiac muscle wt α-actin and its mutants were expressed in the *baculovirus/Sf21*-system [12,52] as detailed in the Supplementary Information. Preparations of myosin subfragment-1 (myosin-S1) from skeletal muscle and bovine cardiac muscle [21], of cardiac tropomyosin (cTm) [36,45] and troponin complex (cTn) [43,53,54], the N-terminal C0C2 fragment of human cardiac MyBP-C [55], and the gelsolin deletion mutant G4-6 [46] were performed with modifications of the published procedures and are detailed for every protein in the Supplementary Materials.

### 4.4. ATPase Assay 

Stimulation of the ATPase activity of α-cardiac or skeletal muscle myosin-S1 activated by human wt cardiac α-actin or the cardiomyopathy inducing mutants was performed at 25 °C using a modified version of the NADH-coupled assay according to [26] in a buffer containing 40 mM HEPES, pH 7.4, 25 mM KCl, 2 mM MgCl_2_, 0.5 mM DTT, 0.2 mM NADH and an ATP regeneration system consisting of 0.05 mg/mL pyruvate kinase, 0.5 mM PEP, and 0.02 mg/mL lactate dehydrogenase (LDH). The reaction was started by the addition of myosin-S1 to a final concentration of 0.5 μM. NADH oxidation was measured by the decrease in absorption at 340 nm (1 μM = 6220 M^−1^ cm^−1^) [56,57] using a spectrophotometer (DU640, Beckman Coulter, Krefeld, Germany). The ATPase rates were determined by linear curve fitting and repeated at least three times for each condition with at least two different c-actin variant purifications. 

### 4.5. DNase I Inhibition Assay 

The DNase I inhibition assay was performed as described [26]. The DNase test solution contained 50 μg/mL salmon sperm DNA (Sigma-Aldrich D1626) in 10 mM Tris-HCl, pH 8.0, 1 mM MgCl_2_, and 0.1 mM CaCl_2_. To determine the endonuclease activity of DNase I, a 10 μL pre-incubation reaction containing 3.2 μM DNase I from bovine pancreas (Sigma-Aldrich DN25) and zero to 6.4 μM of G-actin was prepared in G-buffer and incubated at room temperature for 20 min. Aliquots of the samples were added to 0.8 mL of 50 μg/mL DNA-solution and the absorbance was immediately monitored at 260 nm for 10 min using the Beckman DU 640 spectrophotometer. The DNase I activity was determined from the initial linear rates of OD 260 nm increase and expressed as Kunitz units (KU/min; 1 KU = ΔOD 260 nm of 0.001).

### 4.6. Gel Electrophoresis and Immunoblotting

Polyacrylamide gel electrophoresis in the presence of SDS (SDS-PAGE) was performed as given [58]. Native gel electrophoresis was performed on 10% polyacrylamide gels without SDS and run as described previously [59].

Cells were lysed 10 mM Tris–HCl, pH 7.4, 100 mM NaCl, 1 mM EDTA, 1 mM EGTA, 1 mM NaF, 20 mM Na_4_P_2_O_7_, 2 mM Na_3_VO_4_, 1% Triton X-100, 10% glycerol, 0.1% SDS, 0.5% deoxycholate) and vortexed for 30 s and frozen until use. After thawing, the extracts were vortexed again and centrifuged at 20,817× *g* at 4 °C for 5 min. The protein concentration was estimated according to Bradford [60]. A total of 30 μg of protein extracts were separated on 12.5% SDS-PAGE gels. Proteins were transferred to a nitrocellulose membrane using a wet blotter [61]. Subsequently the membranes were blocked for 1 h in Tris-buffered saline with 1% Tween. Subsequently, the membranes were blocked for 1 h in Tris-buffered saline with 1% Tween-20 (TBS-T) containing 5% non-fat milk powder (blocking solution) and then incubated overnight at 4 °C with primary antibody diluted in blocking solution (goat anti-HA 1:500, mouse anti-cardiac α-actin 1:200, and rabbit anti-actin C11 at 1:1000 dilution). After three washing steps with TBS-T for 15 min at room temperature, the nitrocellulose sheets were incubated with secondary antibodies linked to horse radish peroxidase (HRP) diluted in blocking solution (1:2000) directed against either mouse or rabbit or goat for 1 h at RT. The nitrocellulose membranes were developed with the help of an enhanced chemiluminescence (ECL) system (Biorad, Munich, Germany). Occasionally, the membranes were subsequently stripped, re-blotted, and immunostained for total actin.

### 4.7. Actin Polymerization Assays 

Actin polymerization rates were determined by the increase in fluorescence caused by the incorporation of pyrene-labeled actin into actin filaments [12,27]. Pyrene-labelled actin was pre-cleared by dialysis against G-buffer (10 mM Tris-HCl, pH 8.0, 0.2 mM CaCl_2_, 7 mM β-mercaptoethanol, 1 mM ATP) and centrifugation at 100,000× *g* for 30 min. In these tests, we used pyrene-labelled skeletal muscle actin that was added to the c-actins at a ratio of 20:1 (0.25 to 5 c-actin). Since pyrene-labeled skeletal-actin on its own at 0.25 μM did not show significant polymerization (i.e., increase in fluorescence), we therefore assumed that the increase in fluorescence observed after mixing it with globular c-α-actin in G-buffer was solely due to the polymerization of the c-α-actins. Polymerization was induced by the addition of 2 mM MgCl_2_ and 0.05 M KCl and the subsequent increase in pyrene-fluorescence was monitored with excitation and emission wavelengths of 365 nm and 385 nm, respectively, using a Shimadzu RF5001PC spectrofluorometer.

The critical concentration of c-α-actin polymerization was determined after varying the concentrations of the c-α-actins supplemented with 5% pyrene-actin and polymerized in the presence of 2 mM MgCl_2_ and 0.05 M KCl overnight. The actin concentrations varied from 0.1 to 10 μM. The steady-state fluorescence of polymerized actin was plotted versus monomeric actin concentration and the critical concentration was calculated from the intersections with the abscissa.

### 4.8. Electron Microscopy 

For negative staining F-actin samples were diluted to 0.1 mg/mL and adsorbed to freshly glow-discharged carbon-coated copper grids (200 mesh) for 45 s. Negative staining with 1.0% uranyl acetate was performed as described earlier [57,62]. The samples were examined with a Zeiss electron microscope EM923 (SESAM) run at 120 kV fitted with a TemCamF416 camera (Tietz Video and Image Processing Systems, Gauting, Germany).

### 4.9. Determination of the Ca^2+^-Dependence of Tm Movement on Cardiac F-Actins

The Ca^2+^-dependence of pyrene-labeled cTm movement on polymerized c-actin variants was determined by the increase in the eximer pyrene-fluorescence at excitation and emission wavelengths of 340 nm and 480 nm, respectively, using an Infinite 200 microplate reader (Tecan, Männedorf, Switzerland) [37]. Thin filaments from each c-actin variant were generated by decoration with pyrene-labeled cTm and reconstituted cTn complex, each added at a 1:6 molar ratio to actin subunits. Subsequently, these filaments were further supplemented with myosin-S1 and N-cMyBP-C also at a 1:6 molar ratio to actin subunits. Distinct free Ca^2+^-concentrations in the presence of 1 mM ATP were generated in black 96-well plates [43]. Fluorescence intensities were corrected for background fluorescence and normalized to F_max_ = 1 and F_min_ = 0. Nine experiments were performed for each c-α-actin variant and condition. The data were fitted using a normalized Hill equation (Sigma Plot, Systat Software, Erkrath, Germany). 

### 4.10. Determination of Filaments Length by Fluorescence Microscopy

Freshly purified bovine cardiac actin, recombinant wild-type, and mutant cardiac actins were polymerized in the concentration of 5 μM in 10 mM Hepes buffer pH 7.4 containing 50 mM KCl, 2 mM MgCl_2_, and 0.1 mM CaCl_2_ (Buffer A) The F-actins were stained in the concentration of 1 μM in buffer A containing 2 μM TRITC-labeled phalloidin (SIGMA, Munich, Germany). Immediately and ca. 60 min after staining, the TRITC-phalloidin stained actins ware diluted to 20 to 100 nM in buffer A and 3 μL was dropped on a glass-slide, mixed with 3 μL of a DAKO fluorescence mounting medium (Agilent DAKO, USA/Denmark) then covered with a coverslip.

### 4.11. Imaging Techniques

Fluorescence measurements were performed using a Zeiss Axio Imager Z2m microscope equipped with a Zeiss LD LCI Plan-Apochromat 63×/1.2 multi-immersion objective and Zeiss Axiocam 503 color camera using glycerol as an immersion medium. Rhodamine fluorescence was excited using the LED 555 of the solid-state light source Colibri 7 and the quadruple bandpass filter set 90 HE, both from Zeiss. Images were recorded as gray-scale pictures with the microscope-associated ZEN software. The image size was 1936 × 1460 pixels, and the pixel size was 0.116 µm/pixel.

### 4.12. Analytical Tools

The recorded images were analyzed to obtain the length and number of the filaments using ImageJ and the available plugin Ridge Detection (http://fiji.sc/Ridge_Detection, accessed on 01.11.2021). This plugin is based on the detection algorithm described by Steger [63] to detect ridges and lines. The parameter was selected to ensure that all visible filaments were counted without over- or under-counting, and finally set as the line width: 2.0, sigma: 1.08, lower threshold: 11.0, upper threshold: 12.0, minimum line length: 0.86 (=100 nm), maximum line length: 200. At this setting, actin bundles, which look thicker and brighter than single filaments, are not counted. Data were further analyzed using Microsoft Excel to obtain a histogram of length distribution.

Depending on the filament length and density, about 1000 to 8000 filaments were counted per image. Twenty to 25 images per sample were analyzed, resulting in a total filament count between 30,000 to 150,000 per sample. The data were further analyzed by Microsoft Excel to obtain histograms of the length distributions. As the total count of filaments in each sample was different, the histograms are shown as percentages for comparison.

### 4.13. Data Evaluation

DNA sequences were analyzed in DNAstar Lasergene software (DNASTAR Inc., Madison, WI, USA). Densitometric analysis of bands was performed with the help of the Ultra Quant 6.0 software (Thermo Fisher Scientific, Schwerte, Germany). Graphs were plotted in Excel 2007 (Microsoft) or in Origin 8.5 (OriginLab). In both the myosin-S1 ATPase activity and Ca^2+^-dependence of cTm movement assays, the data are given as mean values (±SEM, standard error of the mean). In addition, the significance of the Ca^2+^-dependency data were analyzed by the Student’s *t*-test and the R-square (R^2^) analysis using Sigma Plot software (Systat, Erkrath, Germany).

## 5. Conclusions

Summarizing the special properties of each c-actin mutant at the protein level, we can conclude the following. (1) The **p.A295S** mutant possesses, at low Ca^2+^-concentrations, a higher Ca^2+^-sensitivity of cTm movement and myosin-S1 ATPase stimulation. It has been proposed that this effect was due to a charge disturbance of the A-triad, since residue 295 is located on a helix opposite the A-triad containing loop [27,32,38], which is a sequence of positively charged residues (K326, K328, and R147) implicated in stabilizing Tm binding in the blocked B-position [20]. Its distortion may ease cTm release from the B-position. Indeed, transfection of an insect cardiac A295S mutant in Drosophila led to signs of HCM. The A295S mutant formed straight and long filaments in Drosophila cardiac tubes and conferred a higher Ca^2+^-sensitivity, supporting the notion that a higher contractility paves the path to HCM [20]. Our results, however, clearly show that the purified p.A295S mutant forms only short filaments, but was able to integrate into normal appearing thin filaments of neonatal rat cardiomyocytes [29], suggesting that its reduced polymerizability might be compensated in intact cardiomyocytes by additional binding proteins, for instance, cTm/cTn (Figure 5 and Table 2) and/or titin. Nevertheless, our data indicate the increased Ca^2+^-sensitivity of the p.A295S mutant may be causative for HCM development. 

(2) An opposite behavior may account for DCM induction by the **p.R312H** mutant, which was characterized by a lower Ca^2+^-sensitivity and a steep Hill coefficient (of 3.9). The p.R312H residue is located in subdomain 3 of actin (Figure 1) and also in close vicinity to the A-triad [6,30]. Furthermore, the neighboring residue D311 forms the strongest electrostatic contact to Tm [30]. It therefore appears that p.R312H strengthens cTm binding to the A-triad region (i.e., to the B-state), leading to reduced Ca^2+^-sensitivity. This might also explain the observation that decoration with cTm/cTn did not further increase its myosin-S1 ATPase stimulatory capacity (Figure S2). Furthermore, the p.R312H mutant possessed a reduced polymerizability, suggesting reduced stability, as reported previously [49] and consequently to a decrease in contractility. Thus, we observed for the p.R312H mutants two properties correlated with DCM, namely reduced Ca^2+^-sensitivity leading to hypo-contractility, and decreased filament stability, probably causing filament disarrays in intact cardiomyocytes. 

(3) Our data demonstrate a slightly lower Ca^2+^-sensitivity at low Ca^2+^-concentrations than recombinant wt c-actin for the DCM **p.E361G** mutant, in line with a reduced contractility typical for a DCM causing mutant. Previous reports have revealed a particular property of the E361G mutant, namely its insensitivity toward the Ca^2+^-ion regulatory effect of cTnI phosphorylation [64]. Beta-adrenergic stimulation leads to phosphorylation of two N-terminal serines of cTnI by protein kinase A (PKA) and reduces the Ca^2+^-sensitivity but increases the rate of relaxation of cardiomyocytes [53,55]. Since recombinant cTnI was used in this study, our data are restricted to its non-phosphorylated state. Nevertheless, our data suggest an altered cTnI binding to p.E361G-actin. The C-terminal part of cTnI (spanning residues 137 to 210 including the inhibitory region [39]) forms an α-helical extension at low Ca^2+^-concentration and binds to three consecutive actin subunits parallel to cTm [39]. The binding affinity of this part of cTnI might be reduced by the exchange of a negatively charge for a neutral residue in p.E361G c-actin. Of note, the p.E361G mutation showed the lowest Ca^2+^-sensitivity of pyrene-cTm movement (pCa_50_ = 7.6) in the absence of myosin-S1 (Table 3). In addition, residue 361 is located within the binding region of myosin heads, cMyBP-C and α-actinin [33,49]. Indeed, the p.E361G mutant is reported to possess a threefold lower affinity to α-actinin [49]. This might lead to a reduction in the stability of the attachment of p.E361G containing filaments to the Z-lines and explains the observation that hearts of transgenic mice expressing the E361G mutant developed contractile dysfunction under stress and signs of DCM [64]. Indeed, after transfection of the p.E361G mutant into neonatal rat cardiomyocytes, we observed its preferential incorporation at the M-line (i.e., the minus ends of the sarcomeric thin filaments), but considerably less incorporation at the Z-line (i.e., at the plus ends) [29,46]. Thus, its reduced affinity to α-actinin could weaken the thin filament attachment to the Z-line and be the cause for the reported filament disarray under mechanical stress [49]. The observed filament disarray could be further aggravated by its tendency to form long filaments, leading to disturbances during sarcomerogenesis. 

Finally, we want to emphasize that we tested the possibility that the cTn-binding Ca^2+^-sensitizer levosimendan might correct the decreased Ca^2+^-sensitivity of the DCM causing c-actin mutants. Our data indeed suggest that levosimendan increased the Ca^2+^-sensitivity of the myosin-S1 ATPase stimulation of the p.R312H and p.E361G mutants. Therefore, this result might open new approaches for the treatment of DCM caused by these or similarly acting c-actin mutants.

In summary, our data demonstrate that each c-actin mutation induces slight biochemical albeit specific alterations of its properties. Within the sarcomere, the functional unit of striated muscle is composed of a large assembly of interacting proteins and metabolites. Therefore, small mutation-specific alterations might influence cardiac development and lead to the progression of cardiomyopathies. Our data indeed demonstrate disparate properties concerning the Ca^2+^-sensitivity and filament length and stability of the cardiac actin mutants causing either HCM or DCM. 

## Figures and Tables

**Table 1:** A. Polymerization parameters (of 5 μM c-actins) and B. critical concentrations of polymerization (Cc) of the cardiac actin variants.

**A. Half Time of Polymerization (t _1/2_) in Min.**				
**Bovine c-Actin**	**Recomb. wt c-actin**	**p.A295S**	**p.R312H**	**p.E361G**
2.7 ± 0.6	2.5 ± 0.3	8.5 ± 0.6	10.8 ± 0.5	1.6 ± 0.2
**B. Critical Concentration of Polymerization (Cc) in μM**				
**Bovine c-actin**	**recomb. wt c-actin**	**p.A295S**	**p.R312H**	**p.E361G**
0.21 ± 0.1	0.2 ± 0.09	0.4. ± 0.3	0.7 ± 0.12	0.2 ± 0.11

**Table 2:** Kinetic parameters of the myosin-S1 ATPase stimulation by the c-actins. Apparent K_M_ and Vmax values of the myosin-S1 ATPase stimulation by the c-actin variants (n.d. = not determined).

c-Actin Variant	Bovine c-Actin	wt Recombinant	p.A295S	p.R312H	p.E361G
**apparent KM (µM)**	0.081	0.165	0.966	0.096	0.075
**+TM/TN** **apparent KM (µM)**	n.d.	0.217	3.31	0.0243	0.173
**Vmax** **(µM ATP hydrol/sec/µM S1)**	0.44	0.334	0.799	0.444	0.641
**+TM/TN Vmax** **(µM ATP hydrol/sec/µM S1)**	n.d.	0.563	4.347	0.267	0.769

**Table 3:** Parameters from Ca^2+^ -ion dependence of myosin-S1 ATPase stimulation. Parameters were derived from experiments depicted in Figure 5. All measurements were performed for each c-actin variant in filamentous form after decoration with cTm and cTn at a ratio of 6:1:1. Hill coefficients, pCa_50_-values, and the R-squared (R^2^) values were calculated by the Sigma Plot Systat software (Erkrath, Germany). N gives the number of experiments. Variations are given as SEM. The fold stimulation was calculated from the ratio of the maximal (max.)/minimal (min.) ATPase activity.

Actin Variant	pCa_50_	Hill Coefficient	Min. ATPase µM ATP hyd/µM S1/s	Max. ATPase µM ATP hyd/µMS1/s	Fold Stimulation (pCa Max./Min.)
Bovine *n* = 3 R^2^ = 0.891	6.93 ± 0.20	1.182 ± 0.54	0.086	0.44	5.625
WT rec *n* = 4 R^2^ = 0.894	6.84 ± 0.09	1.68 ± 0.559	0.093	0.379	4.0
p.A295S *n* = 3 R^2^ = 0.933	7.06 ± 0.09	1.497 ± 0.452	0.078	0.387	4.96
p.R312H *n* = 3 R^2^= 0.789	6.92 ± 0.19	1.342 ± 0.824	0.054	0.346	5.83
p.E361G *n* = 3 R^2^ = 0.927	6.84 ± 0.11	1.021 ± 0.256	0.057	0.301	5.0

**Table 4:** Parameters of the myosin-S1 ATPase stimulation by the DCM c-actin mutants p.R312H and E361G in the presence of 20 µM Levosimendan. Mean values of triplicate experiments. Variations are given as SEM.

	p.R312H	p.R312H + Levo	WTc-Actin	p.E361G	p.E361G + Levo	WT c-Actin
**pCa_50_**	6.923 ± 0.196	7.19 ± 0.20	6.84 ± 0.09	6.84 ± 0.11	7.44 ± 0.34	6.84 ± 0.09
**Hill coefficient**	1.342 ± 0.824	1.9 ± 1.92	1.68 ± 0.55	1.02 ±0.25	1.75 ±1.36	1.68 ± 0.55

**Table 5:** Parameters of the Ca^2+^-ion dependence of fluorescence increase in pyrene-cTm (i.e., cTm movement).

	**Bovine c-Actin**			**Recomb. wt c-Actin**		
	**pCa_50_**		**Hill**	**pCa_50_**		**Hill**
**+cTm/cTn**	7.32 ± 0.14 (*n* = 9; R^2^ = 0.724)		1.03 ± 0.27	7.55 ± 0.21 (*n* = 4; R^2^ = 0.797)		0.88 ± 0.31
**cTm/cTn + S1**	7.53 ± 0.12 (*n* = 6; R^2^ = 0.812)		1.22 ± 0.31	7.45 ± 0.13 (*n* = 4; R^2^ = 0,871)		1.31 ± 0.36
**cTm/cTn + MyBP-C**	7.15 ± 0.14 (*n* = 2; R^2^ = 0.90)		1.52 ± 0.67	7.48 ± 0.17 (*n* = 3; R^2^ = 0.913)		1.74 ± 0.63
**cTm/cTn + S1 + MyBP-C**	7.47 ± 0.2 (*n* = 3; R^2^ = 0.789)		1.1 ± 0.42	7.32 ± 0.06 (*n* = 3; R^2^ = 0.977)		1.58 ± 0.26
	**p.A295S**		**p.R312H**	**p.E361G**		
	**pCa_50_**	**Hill**	**pCa_50_**	**Hill**	**pCa_50_**	**Hill**
**+cTm/cTn**	7.32 ± 0.1 (*n* = 9; R^2^ = 0.873)	1.55 ± 0.43	7.1 ± 0.03 (*n* = 3; R^2^ = 0.950)	3.9 ± 2.4	7.6 ± 0.11 (*n* = 6; R^2^ = 0.90)	1.05 ± 0.2
**cTm/cTn + S1**	7.26 ± 0.61 (*n* = 4; R^2^ = 0.8638)	1.74 ± 0.61	6.76 ± 0.21 (*n* = 5; R^2^ = 0.817)	1.28 ± 0.26	7.07 ± 0.07 (*n* = 6; R^2^ = 0.761)	2.77 ± 1.56
**cTm/cTn +MyBP-C**	7.37 ± 0.1 (*n* = 5; R^2^ = 0.936)	0.85 ± 0.13	7.1 ± 0.09 (*n* = 3; R^2^ = 0.936	1.42 ± 0.37	7.38 ± 0.2 (*n* = 3; R^2^ = 0.879)	0.86 ± 0.27
**cTm/cTn +S1 + MyBP-C**	7.22 ± 0.16 (*n* = 5; R^2^ = 0.842)	0.96 ± 0.25	7.5 ± 0.17 (*n* = 3; R^2^ = 0.893)	1.69 ± 0.65	7.13 ± 0.14 (*n* = 3; R^2^ = 0.878	1.33 ± 0.49

## Data Availability

Data is contained within the article.

## Supplementary Materials

The following supporting information can be downloaded at: https://www.mdpi.com/article/10.3390/ijms23084465/s1. Detailed information can be found in the supplementary materials [12,21,31,37,45,52,54,55,65,66].
