# Supplementary material for: Functional Characterization of Cardiac Actin Mutants Causing Hypertrophic (p.A295S) and Dilated Cardiomyopathy (p.R312H and p.E361G)
Source: Int J Mol Sci. 2022 Apr 18;23(8):4465. doi: 10.3390/ijms23084465 (PMC9024677; doi:10.3390/ijms23084465)
Supplement: Supplementary file 1 [file ijms-23-04465-s001.zip › ijms-1693968-supplementary.pdf]

Supplementary Information

Materials and Methods

Protein expression and purification

Purification of rabbit skeletal and bovine cardiac actin:

Rabbit skeletal muscle and bovine c-actin were prepared from dried acetone powder obtained from fresh rabbit psoas muscle or bovine hearts (obtained from the local slaughterhouse) as described [6]. G-actin was stored in G-buffer (5 mM HEPES-OH, pH 7.4, 0.1 mM CaCl2, 0.5 mM NaN3, and 0.2 mM ATP, pH 7.4) at 4°C or flash frozen in liquid nitrogen after addition of 5% succhrose at stored at -80°C.

Preparation of subfragment 1 from skeletal muscle and bovine cardiac myosin:

The myosin containing fractions from the acetone powder preparation were mixed with glycerol (1:1) and stored at -20°C until use. For preparation of myosin subfragment 1 (myosin-S1) the respective stored myosin was dialysed against low salt buffer to induce myosin precipitation. After centrifugation at 12,000 rpm for one hour the myosin containing precipitate was digested with chymotrypsin [10] for 8 to 10 min. After protease inactivation by addition of 1 mM PMSF the digest was dialysed against 50 mM imidazole, pH 7.0, over night at 4°C. After centrifugation at 12,000 rpm for one hour the supernatant was loaded on a DEAE-column equilibrated 50 mM imidazole-buffer, pH 7.0, and eluted with a salt gradient from 0-150 mM NaCl following the described procedure. The fractions containing pure myosin-S1 identified by SDS-Page were pooled and concentrated by Viva-Spin (Millipore, Witten, Germany). The myosin-S1 concentration was determined photometrically (ε = 11.3 cm-1 mg/ml-1).

Expression and purification of recombinant gelsolin deletion mutant G4-6:

Escherichia coli strain Rosetta (DE3) pLysS (Novagen) was transformed by pCOLDII-G4-6 plasmid and grown in Luria-Bertani medium containing 75 μg/ml ampicillin and 25 μg/ml chloramphenicol to the optical density of 0.6 measured at 600 nm. Protein expression was induced by incubation on ice for 30 min and shaking for 23 h at 20 °C in the presence of 1 mM isopropylthio-D-galactoside (IPTG). Harvested cells from 1 l culture were lysed by sonication (10 x 10 sec) in a binding buffer (10 mM Tris-HCl, pH 8.0, 50 mM KCl, 5 mM $CaCl_2$, 7 mM ß-mercaptoethanol, 1 mM PMSF, 0.01 mg/ml leupeptin, 1 mM ATP) and gently shaken for 45 min at 4°C. After centrifugation at 12,000 x g for 30 min, the sterile filtered supernatant was applied to a Ni^{2+}-affinity column by use of FPLC (AKTA Purifier, GE Amersham). After washing with 6 column volumes low salt buffer (10 mM Tris-HCl, pH 8.0, 50 mM KCl, 5 mM $CaCl_2$) and high salt buffer (10 mM Tris-HCl, pH 8.0, 350 mM KCl, 5 mM $CaCl_2$) gelsolin G4-6 was eluted by an imidazole-gradient (0-1 M, pH 8.0) and the combined G4-6 fractions were dialyzed against 50 mM Tris-HCl (pH 8.0), 50 mM KCl, 1 mM $CaCl_2$ and 1 mM DTT. The protein was concentrated with VIVASPIN 20 concentrator (Sartorius Stedim Biotech, Aubagne, France), supplemented with 3 % sucrose, flash frozen in liquid N2 and stored at -80°C.

Expression and Purification of Recombinant Actin:

The human cardiac muscle wild type α-actin and the mutants were expressed in the *baculovirus*/*Sf21*-system [2]. The preparation of recombinant *baculoviruses* was performed according to the manufacturer´s recommendation (Invitrogen) by transforming the pFastBac Dual vectors into DH10Bac *E.coli* cells, bacmid isolation and transfection into *Sf21* insect cells using Cellfectin II (Invitrogen). Infected *Sf21*-cells were harvested after 3 days and stored at -80 °C.

Purification of tag-free wild-type cardiac α-actin and both mutants was performed by affinity chromatography using the gelsolin deletion mutant G4-6 as described [7, 8], see Figure S1. The cells from 2-5 l culture were lysed by sonication and pipetting in 150 ml of KCl-free lysis-buffer (10 mM Tris-HCl, pH 8.0, 5 mM $CaCl_2$, 20 mM imidazole, 4 % Triton-X, 1 mg/ml Tween-20, 5 mM DTT, and a protease inhibitor cocktail). After addition of purified gelsolin G4-6 (10 mg/ 4 x 107 cells) the

solution was gently stirred at 4°C overnight. After centrifugation at 230,000g for 30 min (OptimaTM LE-80K Ultracentrifuge, BECKMAN COULTER) the supernatant was sterile filtered and applied to a Ni2+-affinity column by use of a FPLC (AKTA Purifier, GE Amersham). The resin was washed with Tris-HCl buffer (10 mM, pH 8.0) including 1 mM $CaCl_2$. Actin was eluted with EDTA-Buffer (10 mM Tris-HCl, pH 8.0, 1.5 mM EDTA) and dialysed against Tris-HCl-buffer (10 mM, pH 8.0) including 0.2 mM $MgCl_2$ and 1 mM DTT. G4-6 was recovered by elution with 1 M imidazole and dialysed against Tris-HCl-Buffer (10 mM, pH 8.0) including 50 mM KCl, 1 mM $CaCl_2$ and 1 mM DTT. The proteins were concentrated with VIVASPIN 20 concentrators supplemented with 3% sucrose, flash frozen in liquid N2 and stored at -80 °C. The different purification steps and the purity of the finally obtained c-actin samples was checked by 12% SDS-Page (as shown in Figure 2 of the main text for wt c-α-ctin and the p.E361G mutant). Western blot analysis of the α-cardiac actins was performed with a monoclonal anti-cardiac actin antibody provided by Progen Biotechnik (Heidelberg, Germany). The nomenclature and numeration of human cardiac α-actin wild-type (wt), and of the mutants p.A295S, p.R312K/H and p.E361G were in accordance to that of the Universal Protein Resource database.

Purification of mammalian cardiac tropomyosin:

Cardiac α-tropomyosin was chromatographically purified from acetone dried powder prepared from pig heart according to [3] and then labelled with pyrene-maleimide under denaturing conditions according to [5].

Expression and purification of human cardiac troponin subunits and troponin reconstitution:

For protein expression, the plasmids encoding wildtype human cTnC and the N-terminal fragment C0C2 of cMyBP-C were transformed into *E.coli* T7 Express lysY cells while the plasmids encoding wild type hcTnI and hcTnT were transformed into *E. coli* BL21 (DE3). The isolation of hcTnC was performed following the method described [1]. Human cTnI was purified according to [9] and hcTnT according to [4]. For the reconstitution of wild type hcTn troponin complex, the individual

References

1. Babu A, Su H, Ryu Y, Gulati J (1992) Determination of residue specificity in the EF-hand of troponin C for Ca2+ coordination, by genetic engineering. J Biol Chem 267:15469–15474. doi: 10.1016/S0021-9258(19)49558-6
2. Bai F, Caster HM, Rubenstein PA, Dawson JF, Kawai M (2014) Using baculovirus/insect cell expressed recombinant actin to study the molecular pathogenesis of HCM caused by actin mutation A331P. J Mol Cell Cardiol 74:64–75. doi: 10.1016/j.yjmcc.2014.04.014
3. Bailey K (1948) Tropomyosin: a new asymmetric protein component of the muscle fibril. Biochem J 43:271–279. doi: 10.1042/bj0430271
4. Deng Y, Schmidtmann A, Redlich A, Westerdorf B, Jaquet K, Thieleczek R (2001) Effects of phosphorylation and mutation R145G on human cardiac troponin I function. Biochemistry 40:14593–14602. doi: 10.1021/bi0115232
5. Graceffa P, Lehrer SS (1980) The excimer fluorescence of pyrene-labeled tropomyosin. A probe of conformational dynamics. J Biol Chem 255:11296–11300
6. Mannherz HG, Brehme H, Lamp U (1975) Depolymerisation of F-actin to G-actin and its repolymerisation in the presence of analogs of adenosine triphosphate. Eur J Biochem 60:109–116. doi: 10.1111/j.1432-1033.1975.tb20981.x
7. Müller M, Mazur AJ, Behrmann E, Diensthuber RP, Radke MB, Qu Z, Littwitz C, Raunser S, Schoenenberger C-A, Manstein DJ, Mannherz HG (2012) Functional characterization of the human α-cardiac actin mutations Y166C and M305L involved in hypertrophic cardiomyopathy. Cell Mol Life Sci 69:3457–3479. doi: 10.1007/s00018-012-1030-5
8. Ohki T, Ohno C, Oyama K, Mikhailenko S V, Ishiwata S (2009) Purification of cytoplasmic actin by affinity chromatography using the C-terminal half of gelsolin. Biochem Biophys Res Commun 383:146–150. doi: 10.1016/j.bbrc.2009.03.144
9. Reiffert S, Maytum R, Geeves M, Lohmann K, Greis T, Blüggel M, Meyer HE, Heilmeyer LMG, Jaquet K (1999) Characterization of the cardiac holotroponin complex reconstituted from native cardiac troponin T and recombinant I and C. Eur J Biochem 261:40–47. doi: https://doi.org/10.1046/j.1432-1327.1999.00261.x
10. Taylor RS, Weeds AG (1976) The magnesium-ion-dependent adenosine triphosphatase of bovine cardiac Myosin and its subfragment-1. Biochem J 159:301–315. doi: 10.1042/bj1590301
